# Stress Reduction through Audio Distraction in Anxious Pediatric Dental Patients: An Adjunctive Clinical Study

**DOI:** 10.5005/jp-journals-10005-1254

**Published:** 2015-02-09

**Authors:** Divya Singh, Firoza Samadi, JN Jaiswal, Abhay Mani Tripathi

**Affiliations:** Assistant Professor, Department of Pedodontics and Preventive Dentistry, Rama Dental College Hospital and Research Centre, Kanpur, Uttar Pradesh, India; Head, Department of Pedodontics and Preventive Dentistry, Sardar Patel Postgraduate Institute of Dental and Medical Sciences, Lucknow, Uttar Pradesh, India; Director, Department of Pedodontics and Preventive Dentistry, Sardar Patel Postgraduate Institute of Dental and Medical Sciences, Lucknow, Uttar Pradesh, India; Reader, Department of Pedodontics and Preventive Dentistry, Sardar Patel Postgraduate Institute of Dental and Medical Sciences, Lucknow, Uttar Pradesh, India

**Keywords:** Audio distraction, Venham's picture test, Pulse oximeter.

## Abstract

**Aim:** The purpose of the present study was to evaluate the eff-cacy of ‘audio distraction’ in anxious pediatric dental patients.

**Materials and methods:** Sixty children were randomly selected and equally divided into two groups of thirty each. The first group was control group (group A) and the second group was music group (group B). The dental procedure employed was extraction for both the groups. The children included in music group were allowed to hear audio presentation throughout the treatment procedure. Anxiety was measured by using Venham's picture test, pulse rate, blood pressure and oxygen saturation.

**Results:** ‘Audio distraction’ was found efficacious in alleviating anxiety of pediatric dental patients.

**Conclusion:** ‘Audio distraction’ did decrease the anxiety in pediatric patients to a significant extent.

**How to cite this article:** Singh D, Samadi F, Jaiswal JN, Tripathi AM. Stress Reduction through Audio Distraction in Anxious Pediatric Dental Patients: An Adjunctive Clinical Study. Int J Clin Pediatr Dent 2014;7(3):149-152.

## INTRODUCTION

Pediatric patients often respond in a bizarre of ways to the dental treatment offered, they may either readily accept dental treatment or may be extremely fearful, stubbornly resistant or reluctant for any form of treatment. The role of pediatric dentist in managing an anxious child is not only to control the ailment with which the child reports but also to teach the child appropriate means to manage anxiety.^1^

One such nonaversive modality to manage a child appropriately in dental clinic is distraction. The disruptive behavior of few pediatric dental patients' can be controlled by diverting their attention and engaging them in alternative activities like watching TV, playing video games, or listening to audio taped music.

The present study is designed in concordance with a novel behavior management technique ‘music distraction’ or ‘audio distraction’. ‘Audio distraction’ is the nona-versive technique in which the patient hears to music throughout a stressful dental treatment procedure.^1,2^

The success of distraction technique in medical settings and adult patients is well documented but the eff-cacy of this technique in dental procedures still needs to be investigated elaborately.^1,2^

Aim of the present study was to evaluate the efficacy of ‘audio distraction’ in anxious pediatric dental patients.

## MATERIALS AND METHODS

**Fig. 1:**
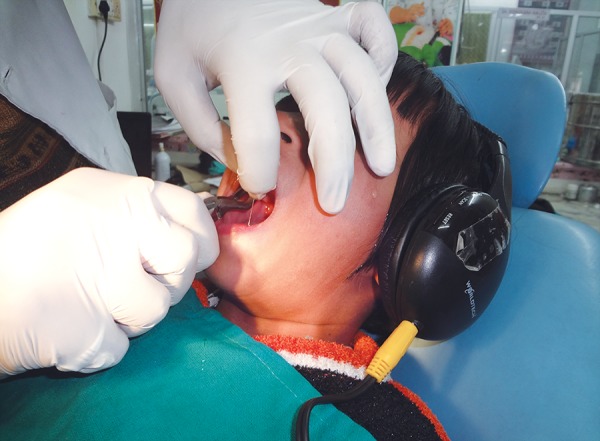
Extraction in audio distraction group

**Fig. 2:**
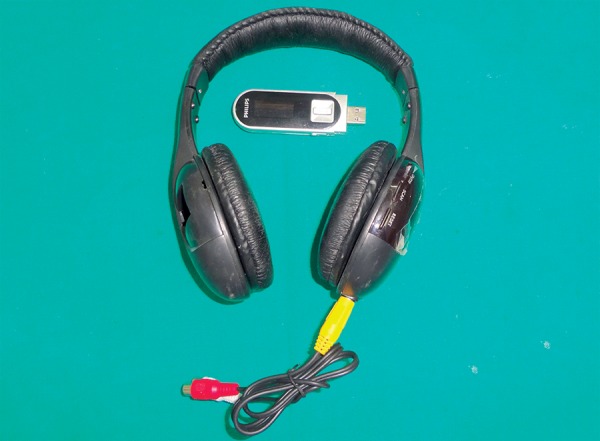
Audio distraction apparatus

**Fig. 3:**
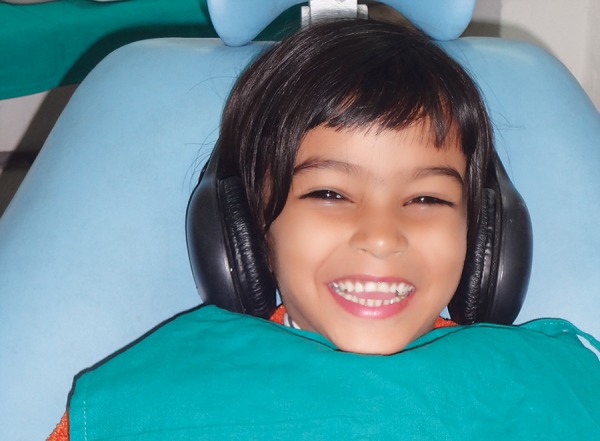
Patient listening to music during the procedure

**Fig. 4:**
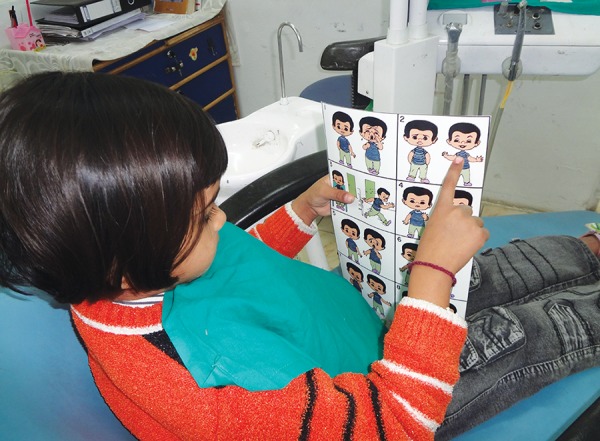
Measurement of child's anxiety with VPT

**Fig. 5:**
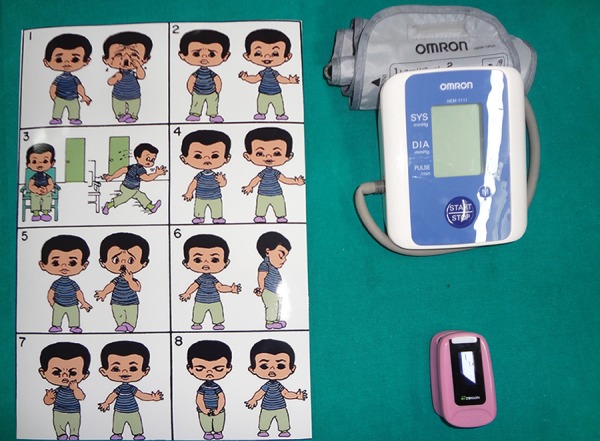
Apparatus–blood pressure machine, pulse oymeter and VPT

**Fig. 6:**
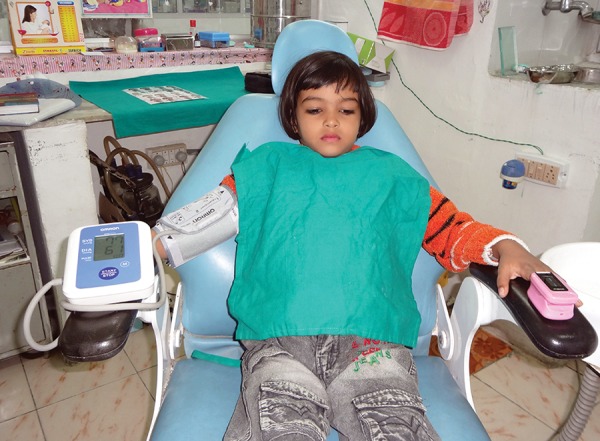
Measurement of pulse rate, oxygen saturation and blood pressure in the patient

Sixty children aged between 6 and 12 years without any previous dental experience were selected for the study. The patients selected were well-oriented to time and space without any mental or physical handicapped. They were randomly and equally divided into two groups of thirty each, i.e. control group (group A) and music group (group B). The dental procedure employed was extraction (Fig. 1). The choice of the type of music was left over patients' will and selection. The patients in the music group listened to the selected audio presentation by head phones throughout the treatment procedure (Figs 2 and 3). The level of child's anxiety was measured using Venham's picture test: a scale for measuring self reported anxiety in children, pulse rate, oxygen saturation and blood pressure (Fig. 4). Both the pulse rate and oxygen saturation was measured using pulse oximeter (Figs 5 and 6).

### Statistical Analysis

The values obtained were statistically analyzed using Students ‘t’ test and ANOVA test; Paired t-test; chi-square test and Wilcoxon signed rank test.

## RESULTS

The present study was carried out at Department of Pedodontics and Preventive Dentistry, Sardar Patel Postgraduate Institute of Dental and Medical Sciences, Lucknow, Uttar Pradesh.

The scores in Table 1 depicts and evaluates the change in pulse rate, systolic blood pressure (SBP), diastolic blood pressure (DBP), oxygen saturation and Venham's picture test (VPT) in control group:

In control group, a change of 5.0 ± 17.4 bpm in pulse rate, 3.8 ± 6.7 mm Hg in SBP, 2.2 ± 7.7 mm Hg in DBP, absolute reduction in number of cases with O_2_ saturation <95% and a mean change of 1.90 ± 1.32 in VPT scores was observed as compared to baseline.

The change in pulse rate, DBP and oxygen saturation was not statistically significant, however, change in SBP and V PT was found to best atistically significant (p < 0.05).

The scores in Table 2 evaluate the change in pulse rate, SBP, DBP, oxygen saturation and VPT in experimental group.

In experimental group, a change of –13.7 ± 12.8 bpm in pulse rate, –3.4 ± 5.7 mm Hg in SBP, –1.5 ± 7.1 mm Hg in DBP, an increase of 2 (6.7%) cases with oxygen saturation <95% and a mean change of –3.00 ± 2.15 in VPT scores was observed as compared to baseline. Except for change in DBP and oxygen saturation all the other changes were found to be statistically significant (p < 0.05).

**Table 1:** **Table 1:** Change in pulse rate, SBP, DBP, oxygen saturation and VPT in control group as compared to baseline

*Sl. no.*		*Variable*		*Baseline*		*At follow-up*		*Change*		*Significance of change*	
1.		Pulse rate*		102.4 ± 15.8		107.4 ± 19.8		5.0 ± 17.4		t = 1.578; p = 0.125	
2.		SBP*		124.0 ± 12.2		127.8 ± 9.5		3.8 ± 6.7		t = 2.988; p = 0.006	
3.		DBP*		81.2 ± 10.8		83.4 ± 8.0		2.2 ± 7.7		t = 1.594; p = 0.122	
4.		O_2_ saturation<95%^@^		3 (10%)		0 (0%)		–3 (10%)		χ^2^ = 3.158; p = 0.076	
5.		VPT^$^		4.43 ± 1.70		6.33 ± 1.45		1.90 ± 1.32		z = 4.174; p < 0.001	

*Paired t-test; ^@^Chi-square test;^$^ Wilcoxon signed rank test, DBP: Diastolic blood pressure, SBP: Systolic blood pressure; VPT: Vibratory perception threshold

**Table 2:** **Table 2:** Change in pulse rate, SBP, DBP, oxygen saturation and VPT in experimental group as compared to baseline

*Sl. no.*		*Variable*		*Baseline*		*At follow-up*		*Change*		*Significance of change*	
1.		Pulse rate*		100.5 ± 15.4		87.1 ± 12.1		–13.7 ± 12.8		t = 5.724; p < 0.001	
2.		SBP*		122.3 ± 12.5		118.8 ± 12.1		–3.4 ± 5.7		t = 3.273; p = 0.003	
3.		DBP*		79.7 ± 12.6		78.2 ± 12.5		–1.5 ± 7.1		t = 1.187; p = 0.245	
4.		O_2_ saturation <95%^@^		1 (3.3%)		3 (10%)		2 (6.7%)		χ^2^ = 1.071; p = 0.301	
5.		VPT^$^		4.83 ± 2.20		1.83 ± 1.68		-3.00 ± 2.15		z = 4.186; p < 0.001	

*Paired t-test; ^@^Chi-square test; ^$^Wilcoxon signed rank test, DBP: Diastolic blood pressure; SBP: Systolic blood pressure; VPT: Vibratory Reception threshold

## DISCUSSION

The aim of the present study was to evaluate the role of ‘audio distraction’ in management of anxious pediatric dental patients during extraction procedure.

Gardner and Licklander first introduced audio analgesia in dental operation for the first time in 1959 (Gardner et al 1959).^3^

The different criteria used in present study to evaluate the anxiety level before and after the treatment procedure was:

*Pulse rate*: Measured by pulse oximeter

*Oxygen saturation*: Measured by pulse oximeter

*Blood pressure*: Measured by BP apparatus Venham's picture test for measuring self reported an x iet y.

Venham's picture test, which is used in the study, is the most reliable measure of self reported. Pulse oximeter which measures pulse rate and oxygen saturation is one of the most acceptable methods for measuring physiological changes. Blood pressure apparatus records blood pressure accurately is another reliable measure to evaluate anxiety level.

Observations from the study indicated that pulse rate in control group was greater than that of music group. Maximum pulse rate during injection phase indicates increase of psychosomatic in origin. Possibly the anticipation of injection provides sympathetic stimulation and catecholamine release which accounts for greater increase in pulse rate (Marwah et al 2005).^2^ They also concluded that the peak anxiety in the last visit of sequential dental visits of patients may be due to the highly stressful event of extraction. In addition to psychological measures, pulse rate is a physiological measurement of anxiety. The increase in pulse rate may be also due to the vasoconstrictor effect of local anesthesia. Oxygen saturation values depicted insignificant variations when compared between both the groups, this was in accordance with findings of Marwah N, Prabhakar AR (2005);^2^ Rayen R (2006);^4^ Korhan EA, Khorshid L, Mehmet M (2011).^5^ Systolic blood pressure was found to be lower in music group than DBP which was not having significant variations between both the groups. This was concurrent with findings of Tse MY, et al.^6^

The observations from the study indicated that VPT gave statistically conclusive results and picture test was very effective measure of judging emotional state of child at chair side. This observation was similar to earlier observations made by Venham et al (1997),^7^ Aitken JC et al.^8^

The results of the present study are consistent with several other studies carried out by Sivakumar N et al. (2010);^9^ Lahmann C et al (2008);^10^ Prabhakar AR et al (2007);^1^ Marwah N et al (2005),^2^ who found that music distraction is an effective means of stress reduction in anxious pediatric dental patients. Whereas, few other studies Aitken JC et al (2002) showed no significant effect of music distraction on anxiety of pediatric dental patients.^8^

## CONCLUSION

Following conclusions were drawn from the study:

Audio distraction did decrease the anxiety in pediatric patients to a significant extent, moreover patients had an overwhelming response to music presentations and wanted to hear them in their subsequent visits.
